# A comprehensive image dataset for the identification of eggplant leaf diseases and computer vision applications

**DOI:** 10.1016/j.dib.2025.111353

**Published:** 2025-01-31

**Authors:** Shakib Howlader, Md. Sabbir Ahamed, Mayen Uddin Mojumdar, Sheak Rashed Haider Noori, Shah Md Tanvir Siddiquee, Narayan Ranjan Chakraborty

**Affiliations:** Multidisciplinary Action Research Lab, Department of CSE, Daffodil International University, Daffodil Smart City, Birulia, Dhaka 1216, Bangladesh

**Keywords:** Plant pathology, Deep learning, Computer vision, Agricultural informatics, Eggplant leaf disease

## Abstract

This dataset on eggplant leaf diseases has been meticulously developed to provide a valuable resource for agricultural research and the advancement of automated disease detection systems. It comprises 4,089 high-resolution images of eggplant leaves, systematically categorized into six distinct classes: Healthy Leaf, Insect Pest Disease, Leaf Spot Disease, Mosaic Virus Disease, White Mold Disease, and Wilt Disease. The images were captured using smartphone cameras under controlled conditions with a consistent white background to ensure clarity and uniformity. To reflect real-world agricultural scenarios, data collection was conducted across multiple geographic locations and in varying lighting conditions. This approach enhances the dataset's diversity and applicability. The dataset underwent thorough manual labelling and preprocessing to ensure accuracy and consistency across all samples. Each image is clearly labelled according to its respective disease class, making the dataset readily usable for machine learning applications. The balanced representation of healthy and diseased leaves allows for comprehensive training and testing of classification models. Designed to support the development of machine learning models for the early detection and classification of eggplant diseases, this dataset holds significant reuse potential in various research domains. It is particularly suitable for applications in plant pathology, precision agriculture, and disease forecasting, where timely and accurate diagnosis is crucial. The dataset is freely available for academic and research purposes, making it a valuable resource for researchers and developers aiming to innovate in agricultural technology and crop management. With its robust design and practical focus, the dataset has the potential to drive advancements in sustainable farming practices and enhance agricultural productivity.

Specifications Table

**Table:** 

Subject	Computer Science.
Specific subject area	Eggplant disease detection, deep learning, image classification, agricultural analytics, and plant health monitoring.
Type of data	Image.
Data collection	The data for this study was collected manually from agricultural fields located in Khagan, Ashulia, Sirajganj, and Madaripur during September and October 2024. The collection process was supervised by an agricultural expert to ensure accurate and comprehensive representation of eggplant leaf conditions. High-resolution images were taken using Poco F3 and Realme 7 smartphones under controlled settings to maintain clarity and uniformity. A total of 4,089 images were captured, covering six distinct categories of healthy and diseased leaves. The dataset includes a diverse range of samples, ensuring variations in lighting, angles, and stages of leaf health. Each image was meticulously labelled to make the dataset suitable for deep learning models focused on plant disease detection.
Data source location	Data was collected from the following locations in Bangladesh: 1. Eggplant field Khagan Bazar, Savar, Dhaka (Latitude: 23°52′35.0″N, Longitude: 90°18′53.3″E) 2. Eggplant field in Ashulia, Savar, Dhaka (Latitude: 23°53′53.1″N, Longitude: 90°19′59.1″E) 3. Eggplant field in Khokshabari, Sirajganj (Latitude: 24°29′22.7″N, Longitude: 89°41′06.0″E) 4. Eggplant field in Char Keshabpur, Shibchar, Madaripur (Latitude: 23°21′32.9″N, Longitude: 90°11′48.5″E) 5. Eggplant field in Daffodil Smart City, Khagan, Ashulia (Latitude: 23°52′37.6″N, Longitude: 90°19′16.2″E).
Data accessibility	Repository name: Mendeley Data. Data identification number: 10.17632/d3ypkphghb.2 Direct URL to data: https://data.mendeley.com/datasets/d3ypkphghb/2 Access the dataset at https://data.mendeley.com/datasets/d3ypkphghb/2 and cite using Data ID 10.17632/d3ypkphghb.2.
Related research article	Eggplant leaf disease detection and segmentation using adaptively regularized multi Kernel-Based FuzzyC-Means and Optimal PNN classifier. [1]

## 1 Value of the Data

• This dataset provides a comprehensive collection of 4,089 high-resolution images of eggplant leaves, categorized into six distinct classes. It supports the scientific community by offering high-quality, labeled data for developing machine learning models aimed at early detection and classification of eggplant diseases [2]. This can contribute to advancements in plant pathology and agricultural technology.
• The dataset allows researchers to test different machine learning architectures, including CNNs and lightweight models like MobileNet. It serves as a reliable benchmark for comparing model performances and exploring novel approaches to disease detection.
• The dataset is designed for reuse in various fields such as computer vision, precision agriculture, and disease forecasting. Researchers can use this dataset to train, validate, and benchmark machine learning models for leaf disease detection or adapt it for related studies on plant health and agricultural data analytics [3].
• This dataset is freely available for academic purposes, making it a valuable resource for teaching, research, and collaborative studies. Students and researchers can use it to explore machine learning concepts, image preprocessing techniques, and data-driven approaches to plant health monitoring.
• By addressing issues in agriculture using advanced data science techniques, this dataset promotes collaboration between plant pathologists, computer scientists, and agricultural researchers to solve critical challenges in crop management and disease control.

## 2 Background

Eggplant (Solanum melongena) is a globally important vegetable crop, valued for its nutritional benefits and widespread culinary applications. However, its production is frequently threatened by diseases caused by fungal, bacterial, and viral pathogens, as well as insect pests, leading to significant yield losses and quality degradation [1,4]. Common diseases, such as Leaf Spot Disease, Mosaic Virus Disease, White Mold Disease, and Wilt Disease, can severely impact crop yield and quality if not identified and managed promptly. The motivation for compiling this dataset arose from the need to support advancements in automated disease detection, which has become increasingly important with the growing adoption of precision agriculture. Traditional methods of disease identification rely on manual inspection, requiring specialized expertise and substantial time, which are often impractical for large-scale farming operations. Machine learning and computer vision technologies offer promising solutions by enabling faster and more accurate analysis, provided they are supported by high-quality datasets that represent the spectrum of disease conditions [5]. This dataset was developed to address the lack of comprehensive, labelled data for eggplant leaf diseases. It includes 4,089 high-resolution images captured under controlled conditions, categorized into six disease classes. This work contributes to the broader goal of advancing AI applications in agriculture and crop management.

## 3 Data Description

The Eggplant Leaf Disease dataset is organized into two primary directories: Classified Images. Each directory contains subfolders corresponding to the six categories of eggplant leaf conditions, ensuring a clear and consistent structure for dataset navigation. The dataset contains the 4,089 original images collected manually during September and October 2024 from agricultural fields located in Khagan, Ashulia, Sirajganj, and Madaripur. Images were captured in JPG format using high-resolution cameras under the supervision of agricultural experts. Each image was meticulously labeled into one of six predefined categories: Healthy Leaf, Insect Pest Disease, Leaf Spot Disease, Mosaic Virus Disease, White Mold Disease, or Wilt Disease [1].

**Fig. 1:**
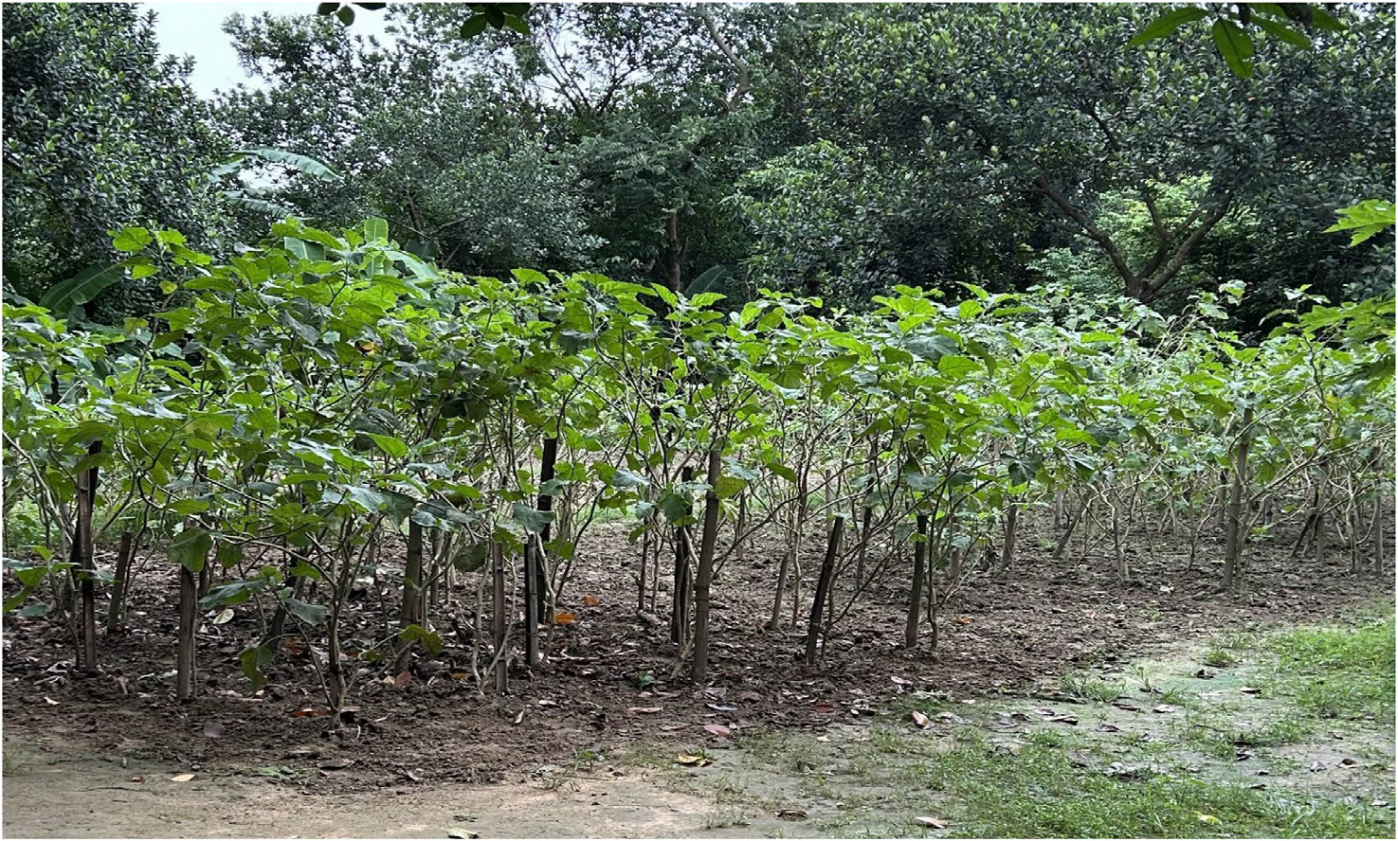
The Real eggplant garden where images in the dataset were collected.

Fig. 1: illustrates a representative field image from one of the data collection sites.

Each class contains several eggplant leaf diseases, along with healthy classes. This will be very important when working on the development of effective models for machine learning in disease detection, hence contributing toward the main goal of improving agriculture in general.

From a better understanding, Table 1 summarizes the six specific classes of eggplant leaf conditions represented in the collected images. Following is the actual class description included in the dataset:

**Table 1:** Overview of diseases by eggplant leaf class.

Name of Class	Description	Visualization
Healthy Leaf	Healthy eggplant leaves are vibrant green, smooth, and free of spots, discoloration, or deformities. They serve as a baseline for disease detection studies, helping train machine learning models to accurately distinguish healthy from diseased conditions [1].	
Insect Pest Disease	Insect pest-affected eggplant leaves display damage such as holes, bite marks, discoloration, or wilting, making them vital for training machine learning models to detect pest infestations and support pest management [6].	
Leaf Spot Disease	Leaf Spot Disease on eggplant leaves is marked by small, round or irregular brown to black spots, often with a halo or discolored edge. These spots can spread, weakening the leaf and potentially causing leaf drop. Accurate identification of these spots is essential for training machine learning models to detect and classify the disease effectively.	
Mosaic Virus Disease	Mosaic Virus Disease in eggplant leaves causes mottled green, yellow, or white patches, often with curling or distortion. These distinct symptoms are crucial for training machine learning models to detect and classify the disease effectively.	
White Mold Disease	White Mold Disease in eggplant leaves features white, cotton-like fungal growth with yellowing, browning, and wilting, essential for training machine learning models to detect the disease.	
Wilt Disease	Wilt Disease in eggplant leaves causes drooping, yellowing, and eventual browning due to disrupted water flow in the plant. These symptoms are crucial for training machine learning models to identify wilt disease accurately.	

This dataset supports multiple advancements in agricultural research, particularly through the integration of automation and machine learning. Models trained on this dataset can detect diseases early by analyzing subtle changes in leaf color, texture, and shape [6]. This allows farmers to minimize crop loss and improve yields. Additionally, the dataset promotes the development of automated grading systems for quality assurance and enables the integration of remote sensing technologies, such as UAVs and satellite systems, for large-scale monitoring [7,6].

By reflecting natural conditions with real-world field images, this dataset enhances the reliability of machine learning models across diverse environments. Covering multiple disease types, it supports precision farming and contributes to sustainable agricultural practices [1,6].

### 3.1 Dataset comparison

We compared our dataset to an existing dataset by Hasan et al. (2023) [8], which focuses on eggplant leaf disease classification. Table 2 provides a detailed comparison between the two datasets, highlighting several key differences that demonstrate the advantages of our dataset. The dataset by Hasan et al. [8] consists of only 392 images. In comparison, our dataset is significantly larger, containing 4,089 high-resolution images divided into the same six classes. The distribution of images in each class is more extensive in our dataset, with notable improvements in categories such as Healthy (1,451 images compared to 67), Mosaic Virus (1,362 images compared to 36), and White Mold (63 images compared to 8).

**Table 2:** Comparison with available datasets of eggplant.

Features	Our Dataset	Hasan et al. [8]
Total Original	4089 images	392 images
Healthy Leaf Class	1451 images	67 images
Insect Pest Class	546 images	94 images
Leaf Spot Class	602 images	111 images
Mosaic Virus Class	1362 images	36 images
White Mold Class	63 images	8 images
Wilt Class	65 images	62 images
Image Clarity	High-resolution, noise free image	Moderate clarity, non-uniform
Background	White, standardized	Natural, inconsistent
Image Size	Uniform size	Non-uniform size
Image Clarity	High-resolution, noise free image	Moderate clarity, non-uniform

In terms of image quality, our dataset stands out with high-resolution, noise-free images captured under controlled conditions with a white, standardized background and uniform size. This ensures consistency and removes potential biases caused by varying backgrounds or image dimensions, as seen in the dataset by M. Hasan, Ava, et al., which features moderate clarity, non-uniform image sizes, and natural, inconsistent backgrounds. Our dataset is designed to support the development of robust machine learning models by providing a balanced and high-quality collection of images across all disease classes. The standardized approach ensures the dataset is ready for immediate use in training AI models for eggplant disease classification.

This contribution aims to improve agricultural practices and disease management, especially in regions where eggplant is a critical crop. Table 2 illustrates the comparison between our dataset and an existing dataset for eggplant leaf disease detection.

Our data collection took place over September and October 2024. To ensure diversity, we captured images of eggplant leaves on different days and at various times throughout the day. The collection sites were carefully selected to represent a range of environments. During the process, we documented essential parameters such as date, weather conditions, time, temperature, humidity, the devices used for image capture and location. A detailed summary of these parameters is provided in Table 3.

**Table 3:** Collection details of eggplant leaf dataset.

Date	Weather	Time	Temperature (°C)	Humidity (%)	Camera Device	Location
17 September 2024	Sunny	Morning	26°C	87 %	Poco F3 (50 %) and Realme 7 (50%)	Khagan Bazar, Savar, Dhaka
24 September 2024	Windy	Afternoon	28°C	86 %	Poco F3 (50 %) and Realme 7 (50%)	Khagan Bazar, Savar, Dhaka
02 October 2024	Cloudy	Afternoon	25°C	75 %	Poco F3 (60 %) and Realme 7 (40%)	Ashulia, Savar, Dhaka
12 October 2024	Sunny	Noon	31°C	86 %	Realme 7 (100%)	Khokshabari, Sirajganj
20 October 2024	Sunny	Noon	30°C	79 %	Poco F3 (100%)	Keshabpur, Shibchar, Madaripur
28 October 2024	Cloudy	Afternoon	27°C	70 %	Poco F3 (40 %) and Realme 7 (60%)	Daffodil Smart City, Khagan, Ashulia

Our dataset was collected from specific regions in Bangladesh, which may limit its generalizability to other regions with different environmental conditions. This constraint could affect its applicability in broader agricultural contexts. To address this limitation in future work, additional data could be collected from diverse geographic regions with varying climates and farming practices. Incorporating data from multiple locations would enhance the dataset's representativeness and utility, making it more applicable to a wider range of agricultural settings.

Currently, our dataset includes six disease classes, which may not cover all diseases affecting eggplants in different regions. Expanding the dataset in the future to include additional diseases from diverse locations will improve its comprehensiveness and applicability to broader agricultural contexts.

While our dataset focuses on capturing static images of visible symptoms, it does not include information on disease progression over time, limiting its effectiveness for early-stage disease detection where signs are often microscopic or biochemical. Future work could explore advanced imaging techniques, such as hyperspectral or thermal imaging, to address this limitation, enhance early detection capabilities, and incorporate longitudinal data to track disease development, providing valuable insights for disease forecasting and management strategies.

## 4 Experimental Design, Materials and Methods

### 4.1 Experimental design

Diseases in crop leaves can severely impact agricultural productivity, reducing yields and farmers' income. Accurate detection of these diseases requires advanced computational methods. This study focuses on diagnosing eggplant leaf diseases using a curated dataset to assist agricultural professionals in effectively identifying and managing these issues [4]. Images were collected from eggplant fields, capturing diverse disease symptoms under various environmental and climatic conditions like various temperature, humidity and time.

The dataset was classified into specific categories: Healthy Leaf, Mosaic Virus Disease, White Mold Disease, Wilt Disease, Insect Pest Damage and Leaf Spot Disease, providing a comprehensive visual resource for disease detection. By following this structured methodology, the dataset becomes a vital tool for developing and validating machine learning models for eggplant leaf disease detection, contributing to better crop monitoring, timely interventions, and sustainable farming practices [9].

**Fig. 2:**
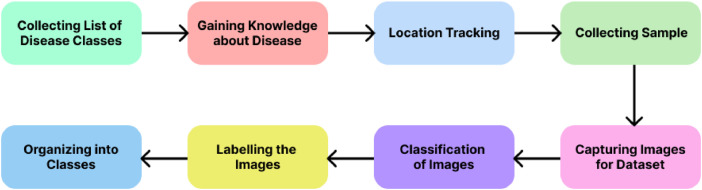
Workflow of the experimental design for the detection and classification of eggplant leaf disease.

In Fig. 2 the workflow for the experimental design begins with collecting a list of disease classes, followed by gaining detailed knowledge about the identified diseases. The next step involves location tracking to pinpoint regions for sample collection. After collecting the samples, images are captured in controlled conditions to ensure consistency. The captured images are then classified into respective categories, labeled appropriately, and organized into distinct classes. This structured workflow ensures the dataset's accuracy and reliability, making it suitable for detecting and classifying eggplant leaf diseases.

### 4.2 Materials (Camera specification)

The Realme 7 features a 64 MP quad-camera with an f/1.8 aperture, ultra-wide 119-degree lens, and macro capabilities for high-resolution, detailed images. It includes AI scene recognition and a 16 MP front camera with HDR and Super Nightscape mode for clear photos in various lighting conditions. The Poco F3 has a 48 MP triple-camera system with an f/1.8 aperture, ultra-wide lens, and macro functionality for crisp and vibrant images. Its 20 MP front camera offers AI beautification and night mode. With 4K video recording and EIS stabilization, it ensures high-quality photography and videography.

### 4.3 Methods

**Fig. 3:**
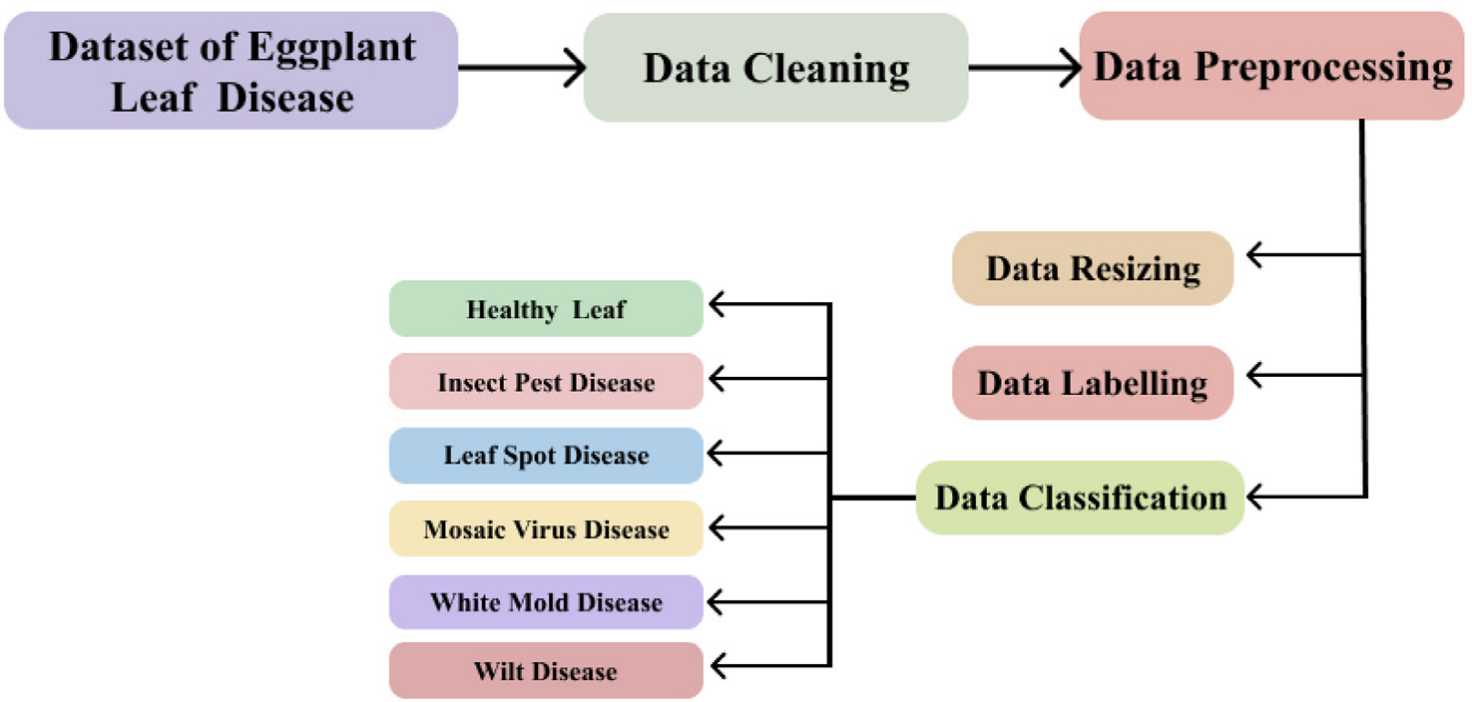
Pre-processing stages of the proposed deep learning model in the detection of eggplant leaves diseases.

The raw images gathered from eggplant fields were processed through several critical steps to prepare them for training deep learning models, as outlined in Fig. 3. Initially, data cleaning was carried out to remove irrelevant, duplicate, or low-quality images, ensuring that only high-quality samples remained suitable for training. Following this, all images were resized to a uniform resolution, ensuring consistency across the dataset. Each image was then labeled into one of six predefined categories: Healthy Leaf, Insect Pest Disease, Leaf Spot Disease, Mosaic Virus Disease, White Mold Disease, or Wilt Disease, creating an organized and structured dataset for machine learning. The pre-processing pipeline ensured the dataset was thoroughly optimized for accurate and reliable model training and evaluation, equipping it to handle real-world challenges effectively [10].

### 4.4 Preprocessing steps

The following preprocessing steps were implemented:

• **Background Removal:** The background of each image was removed and standardized to a white background to eliminate distractions and ensure uniformity across the dataset.
• **Image Resizing:** All images were resized to a uniform dimension to maintain consistency and compatibility with machine learning models.
• **Labeling:** Each image was manually labeled based on the identified disease classes to ensure accurate annotation and facilitate classification tasks.
• **Classification:** Images were organized into six predefined classes: Healthy Leaf, Insect Pest, Leaf Spot, Mosaic Virus, White Mold, and Wilt, forming a structured dataset ready for analysis.

### 4.5 Code used for data preprocessing

GitHub Repository name: Data_Preprocessing

Direct URL of Code: https://github.com/paradoxicalProfessor/Data_Preprocessing

## Limitations

The Eggplant Leaf Disease dataset has some limitations. It was collected from specific regions in Bangladesh, which may limit its applicability to other environments. Our dataset includes only six disease classes, which may not represent all eggplant diseases in different regions. Some disease classes, like White Mold Disease and Wilt Disease, have fewer samples, leading to class imbalances that could affect model performance. The dataset relies on smartphone cameras, which, while effective, lack the diversity of imaging devices. Additionally, it focuses on visible symptoms, excluding early-stage or non-visible signs, which may restrict its use for early detection. The dataset does not include information on disease progression over time, which could provide valuable insights for forecasting.

## Ethics Statement

The authors confirm that the current work complies with the ethical requirements for publication in Data in Brief. This study does not involve human subjects, animal experiments, or data collected from social media platforms. All data were collected ethically, with permissions obtained from field owners where the images were captured. The dataset is publicly available and adheres to the principles of open access research.

## CRediT authorship contribution statement

**Shakib Howlader:** Conceptualization, Methodology, Data curation, Visualization. **Md. Sabbir Ahamed:** Methodology, Data curation, Visualization, Writing – review & editing. **Mayen Uddin Mojumdar:** Methodology, Writing – review & editing, Supervision. **Sheak Rashed Haider Noori:** Visualization. **Shah Md Tanvir Siddiquee:** Investigation. **Narayan Ranjan Chakraborty:** Investigation.

## Data Availability

Mendeley DataEggplant Leaf Disease Detection Dataset (Original data).
